# A Safer Sodium‐Ion Battery Based on Nonflammable Organic Phosphate Electrolyte

**DOI:** 10.1002/advs.201600066

**Published:** 2016-04-23

**Authors:** Ziqi Zeng, Xiaoyu Jiang, Ran Li, Dingding Yuan, Xinping Ai, Hanxi Yang, Yuliang Cao

**Affiliations:** ^1^ College of Chemistry and Molecular SciencesWuhan UniversityWuhan430072P.R. China; ^2^ School of Measurement and Control Technology and Communication EngineeringHarbin University of Science and TechnologyHarbin150080P.R. China

**Keywords:** alloy anode, nonflammable electrolyte, safety, sodium‐ion battery, trimethyl phosphate

## Abstract

Sodium‐ion batteries are now considered as a low‐cost alternative to lithium‐ion technologies for large‐scale energy storage applications; however, their safety is still a matter of great concern for practical applications. In this paper, a safer sodium‐ion battery is proposed by introducing a nonflammable phosphate electrolyte (trimethyl phosphate, TMP) coupled with NaNi_0.35_Mn_0.35_Fe_0.3_O_2_ cathode and Sb‐based alloy anode. The physical and electrochemical compatibilities of the TMP electrolyte are investigated by igniting, ionic conductivity, cyclic voltammetry, and charge–discharge measurements. The results exhibit that the TMP electrolyte with FEC additive is completely nonflammable and has wide electrochemical window (0–4.5 V vs. Na/Na^+^), in which both the Sb‐based anode and NaNi_0.35_Mn_0.35_Fe_0.3_O_2_ cathode show high reversible capacity and cycling stability, similarly as in carbonate electrolyte. Based on these results, a nonflammable sodium‐ion battery is constructed by use of Sb anode, NaNi_0.35_Mn_0.35_Fe_0.3_O_2_ cathode, and TMP + 10 vol% FEC electrolyte, which works very well with considerable capacity and cyclability, demonstrating a promising prospect to build safer sodium‐ion batteries for large‐scale energy storage applications.

## 1 Introduction

Sodium‐ion batteries (SIBs) have recently attracted increasing attention as an alternative to lithium‐ion batteries for large‐scale energy storage applications due to their low cost and natural abundance. Similar to their Li counterparts,^1^ though sodium‐based system has similar electrochemical reaction characteristics compared to lithium‐based one, the larger ionic radius for sodium ion cause sluggish kinetics and volume change during Na storage, leading to lower capacity, poor cycling and rate properties of the Na storage materials. Recently, major efforts have been devoted to promote the electrochemical performance of Na storage materials, for example, Na*_x_*MO_2_,^2^, ^3^, ^4^, ^5^, ^6^, ^7^, ^8^, ^9^, ^10^ polyanionic framework compounds,^11^, ^12^, ^13^, ^14^, ^15^, ^16^, ^17^, ^18^, ^19^ hexacyanoferrate,^20^, ^21^, ^22^, ^23^, ^24^, ^25^, ^26^, ^27^ for the cathode materials, and hard carbons,^28^, ^29^, ^30^, ^31^, ^32^, ^33^ alloys,^34^, ^35^, ^36^, ^37^, ^38^, ^39^, ^40^, ^41^ oxides,^42^, ^43^ sulfides^37^, ^44^, ^45^, ^46^ for the anode materials.

Although the present works on SIBs mainly focus on the Na storage materials, the safety of SIBs is also a critical obstacle for their large‐scale applications because of the flammable nature of their organic carbonate electrolytes and carbon anode used currently, similar to the lithium‐based system. In fact, sodium has much higher chemical activity than lithium though sodium has slightly higher redox potential than lithium (−2.71 V for Na while −3.0 V for Li, vs. SHE). This means that sodium ion batteries have a higher risk for thermal runaway, firing, or explosion in air, causing even more serious accidents than lithium‐ion systems. Therefore, the safety of SIBs is also a critical issue for the large‐scale applications.

To solve the safety issue, it is a most convenient and effective approach to develop safer electrolytes with nonflammability, thermal‐stability, and electrochemical stability. However, previous works are mostly devoted on ionic liquid and solid‐state electrolytes, while few studies have been reported on nonflammable liquid electrolyte for SIBs. NaFSI‐C_1_C_3_pyrFSI electrolyte is report to have good compatibility with a practical anode material such as hard carbon at 90 °C.^47^ And the cathode material (NaFePO_4_) is also discussed in butylmethylpyrrolidinium−bis(trifluorometh‐anesulfonyl)imide (BMP−TFSI) ionic liquid, which performed a capacity of 152 mAh g^−1^ at 75 °C.^48^ A promising Na^+^ ionic conductor based on crystalline NASICON compounds is reported to exhibit an initial discharge capacity of 330 and 131 mA h g^−​1^ for a hard carbon anode and a NaFePO_4_ cathode at a 0.2C‐rate of room temperature.^49^ Although they are safe for battery applications, they have low ionic conductivity at room temperature, difficultly meeting the requirements for large‐scale energy storage.

Consequently, it is very necessary to develop nonflammable liquid electrolytes with comparable ionic conductivity to the current organic carbonate electrolytes. Compared to ionic liquid and solid‐state electrolytes, organic phosphates have lower viscosity, wide liquid temperature range, strong salt‐dissolving ability, and particularly nonflammability, which have been used as a flame‐retardant additive or co‐solvent for lithium‐ion batteries.^50^, ^51^, ^52^, ^53^, ^54^, ^55^, ^56^, ^57^, ^58^, ^59^ However, nonflammable phosphate‐based electrolytes have never been studied for sodium‐ion batteries. Among all the phosphates, trimethyl phosphate (TMP) is the most appropriate choice for safe electrolyte, because of its low viscosity (0.02257 P),^60^ high dielectric constant (21.6), wide liquid temperature range (−46–197 °C)^61^ and chemical stability. But the fatal flaw is that TMP can decompose electrochemically on carbonaceous anode in Li ion electrolytes, failing to form a stable solid electrolyte interphase (SEI) film on the carbon surface.^59^ This problem may also be an obstacle for constructing nonflammable sodium‐ion batteries.

Taking into account the safe hazards due to the flammability of organic carbonate electrolytes and metal Na deposition at low potential in currently developed carbon anodes, we propose a safer sodium ion battery based on TMP‐based nonflammable electrolyte, using NaNi_0.35_Mn_0.35_Fe_0.3_O_2_ cathode and Sb‐based anode. With the addition of fluoroethylene carbonate (FEC) as a solid electrolyte interphase (SEI)‐forming additive, the TMP‐based electrolyte shows an excellent electrochemical compatibility with the Sb‐based anode and NaNi_0.35_Mn_0.35_Fe_0.3_O_2_ cathode, thus rendering the nonflammable batteries with similar capacity and cyclability to conventional Na‐ion batteries using carbonate electrolytes. To our best knowledge, such a nonflammable system has not been reported in sodium ion battery so far.

## 2 **Results and Discussion**

### 2.1 **Physical and Electrochemical Characterizations of TMP‐Based Nonflammable Electrolyte**

**Figure 1:**
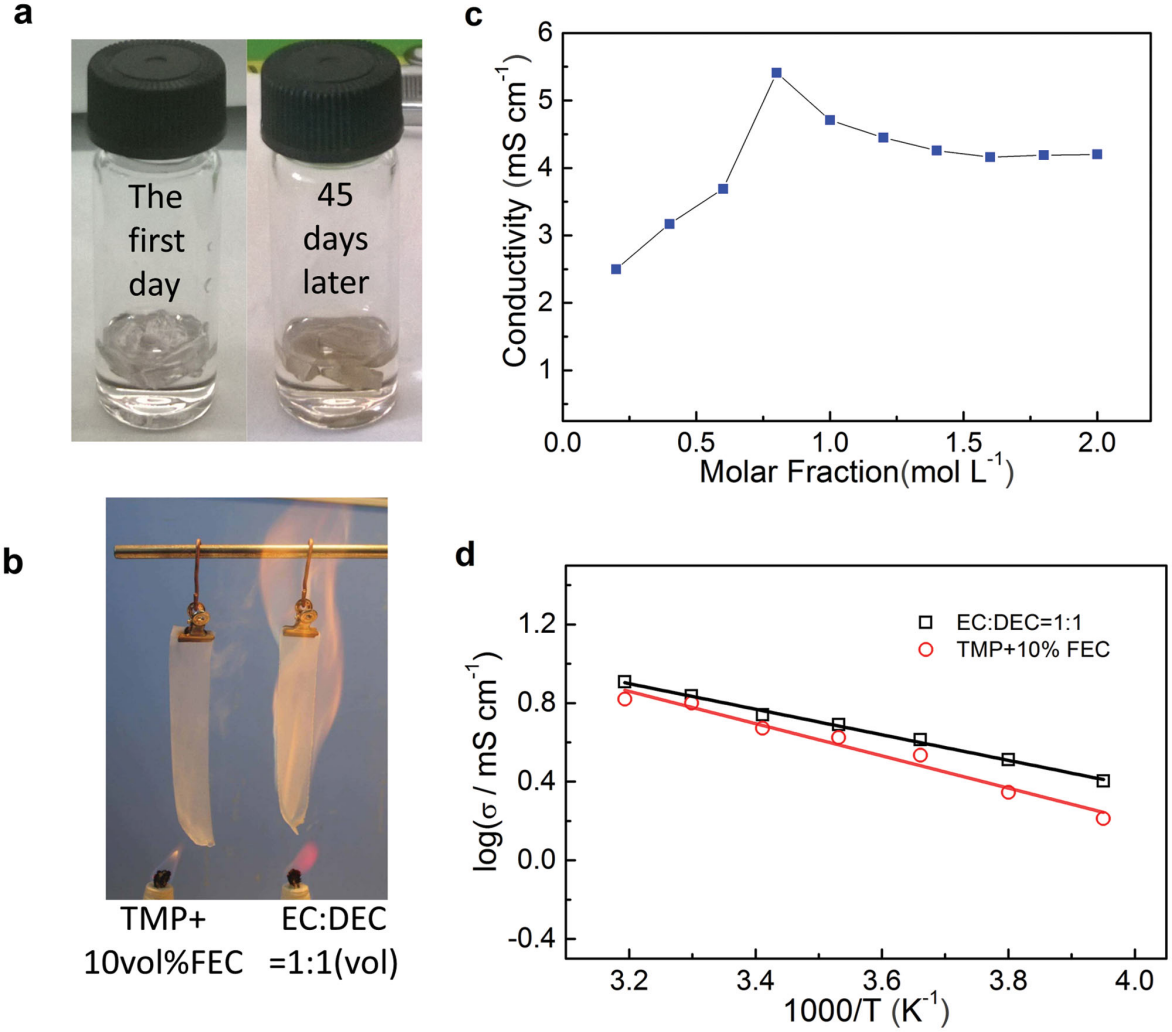
a) Room temperature storage behaviors of the TMP + 10 vol% FEC electrolyte. b) Combustion behaviors of the TMP electrolyte and carbonate electrolyte. c) Concentration dependences of the ionic conductivities of the NaPF_6_/TMP + 10 vol% FEC electrolyte at room temperature. d) Temperature dependences of the ionic conductivities of 0.8 m NaPF_6_/TMP + 10 vol% FEC electrolyte. The ionic conductivity of 1 m NaPF_6_ EC/DEC (1:1) electrolyte is also shown for comparison.

The stability of the TMP‐based electrolyte to metal Na is shown in **Figure** **1**a. It can be apparently observed that after metal Na is stored in the TMP + 10 vol% FEC over 45 d, only the surface of the metal Na becomes a little dim, while the electrolyte is as clear as the fresh one, implying that the very good compatibility of TMP with Na. In order to verify nonflammability of the TMP + 10 vol% FEC electrolyte, a direct igniting experiment is carried out. Figure 1b compares the flammability of the TMP + 10 vol% FEC electrolyte and carbonate electrolyte. It reveals that the TMP + 10 vol% FEC electrolyte cannot be ignited completely even in fire, while the carbonate electrolyte burns up very quickly once ignited. This result shows excellent fire retardancy of the TMP‐based electrolyte.

Another important property of an electrolyte solvent for sodium batteries is the ability to dissolve sodium salt. In our experiment, it is found that TMP solvent can dissolve up to 1.6 mol L^−1^ NaPF_6_, forming considerably conductive electrolyte. Figure 1c gives the room temperature ionic conductivity of NaPF_6_/TMP + 10 vol% FEC electrolyte at various concentrations. It shows that in a wide concentration range of 0.5–2 mol L^−1^ NaPF_6_ electrolyte, the ionic conductivity of the TMP solution can reach a considerable high value of >3 mS cm^−1^. The conductivity increases with NaPF_6_ content, reaches a maximum value of 5.41 mS cm^−1^ at 0.8 mol L^−1^. The reason for the decreased conductivity at high concentration is usually due to the high viscosity of the concentrated electrolyte. Figure 1d shows the ionic conductivities of the NaPF_6_/TMP + 10 vol% FEC and conventional carbonate electrolyte under different temperatures. The ionic conductivity of 0.8 mol L^−1^ NaPF_6_/TMP + 10 vol% FEC electrolyte reaches 5.41 mS cm^−1^ at room temperature (25 °C), which is a little lower than that of conventional carbonate electrolytes (6.44 mS cm^−1^), but it is still able to meet the needs of the majority of the cases. Based on the Arrhenius equation, the activation energy of the TMP‐based electrolyte was calculated to be 2.03 kJ mol^−1^, slightly higher than that of the organic carbonate electrolyte (1.83 kJ mol^−1^). This result implies that the TMP‐based electrolyte has slightly higher temperature dependence than carbonate electrolyte, that is, as the temperature drops, the solubility of NaPF_6_ in the TMP‐based electrolyte decreases much more rapidly than in the carbonate electrolyte.

**Figure 2:**
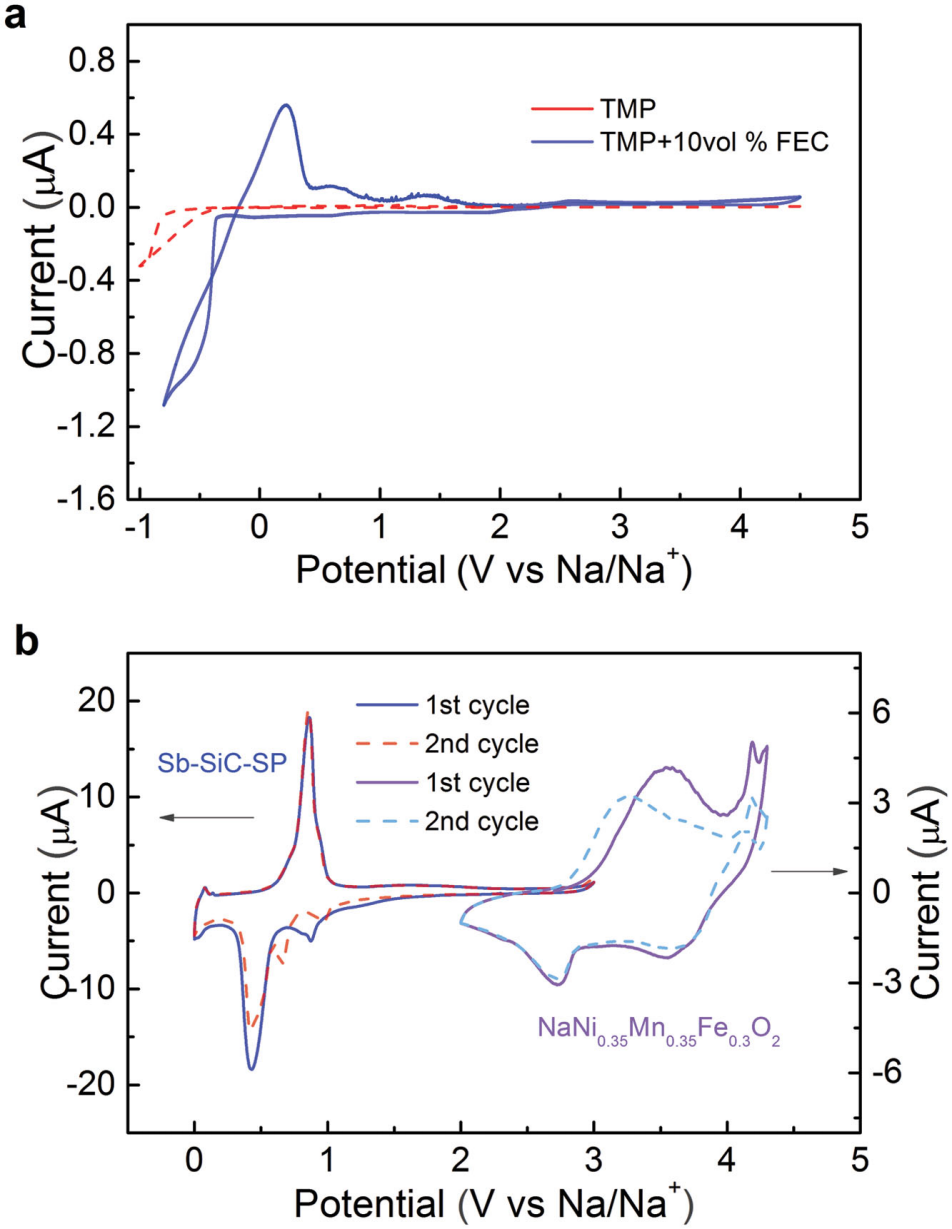
Cyclic voltammograms of a) 0.8 m NaPF_6_/TMP electrolyte with or without 10 vol% FEC and b) the Sb‐based anode and NaNi_0.35_Mn_0.35_Fe_0.3_O_2_ cathode materials in 0.8 m NaPF_6_/TMP + 10 vol% FEC.

To examine the electrochemical stability of the TMP‐based electrolyte, the potential window is measured by cyclic voltammetry (CV) technology on the Pt microelectrode. As shown in **Figure** **2**a, on the whole scan potential range between 0 and 4.5 V, only a pair of the oxidation/reduction peaks appears around 0 V (vs. Na/Na^+^) in the TMP + 10 vol% FEC electrolyte, corresponding to the reversible plating/dissolution reactions of sodium. The CV evidence indicates that the TMP‐based electrolyte has a wide electrochemical window up to 4.5 V (vs Na/Na^+^), which is sufficiently wider than the working potentials of the most of cathode and anode materials currently developed for sodium ion batteries. It should be noted that Na only shows reversible plating/dissolution in TMP + 10 vol% FEC electrolyte. Whereas, no oxidation peak can be observed in blank TMP electrolyte (Figure 2a), revealing the important role of FEC as film formation component to ensure the reversibility of anode materials

The electrochemical compatibility of the TMP‐based electrolyte to the cathode and anode materials is also investigated by CV curves. Figure 2b displays the CV characteristics of the NaNi_0.35_Mn_0.35_Fe_0.3_O_2_ cathode and Sb‐based anode in the TMP + 10 vol% FEC electrolyte. For the Sb‐based anode, it can be seen that during the first negative scan, a weak reduction peak occurs at ≈1.0 V and disappears at the subsequent scans, corresponding to the irreversible decomposition of FEC to form SEI films. A pair of strong reduction/oxidation peaks is observed at 0.5 V and 1.0 V, which can be assigned to the alloying reaction of Sb with Na. Another pair of the weak peaks around 0 V should be attributed to the intercalation/deintercalation reaction of Na in carbon. At the subsequent scans, except for the disappearance of the irreversible current peak at ≈1.0 V due to the formation of SEI film, the CV curves show an additional reduction peaks at 0.63 V, which is apparently attributed to the first‐step alloying reaction of Sb with Na.^36^, ^62^, ^63^ This phenomenon suggests an activation process occurring during first discharge to accelerate the kinetics of the alloying reaction. For the NaNi_0.35_Mn_0.35_Fe_0.3_O_2_ cathode, the CV curves show two pairs of reversible oxidation/reduction peaks appearing at 3.5/2.8 V and 4.25/3.6 V, corresponding obviously to the redox reactions of Ni^2+/3+^‐Fe^3+/4+^ and Ni^3+/4+^, respectively. In fact, the CV features of both the NaNi_0.35_Mn_0.35_Fe_0.3_O_2_ cathode and Sb‐based anode in the TMP + 10 vol% FEC electrolyte are very similar to those in carbonate electrolyte,^63^ suggesting that the TMP‐based electrolyte with FEC additive has no adverse impact on the electrochemical reactions of the Sb‐based anode and NaNi_0.35_Mn_0.35_Fe_0.3_O_2_ cathode.

### 2.2 **Electrochemical Behaviors of Sb‐Based Anodes in the TMP‐Based Electrolyte**

It has been reported that organic phosphates are difficult to form stable SEI film on the surface of carbonaceous anodes even with film‐forming additive.^58^, ^59^ Similar to the lithium‐ion system, TMP also shows strong irreversible decomposition reaction on the surface of hard carbons even with the presence of FEC additive (Figure S1, Supporting Information), that the initial coulombic efficiency of hard carbon is only 40% (Figure S1b, Supporting Information) and there is no obvious redox peak current corresponding to the intercalation and deintercalation of Na^+^(Figure S1a, Supporting Information). Thus, phosphate‐based electrolytes are also not suitable for carbonaceous Na anodes. In fact, since the intercalation potential of Na in hard carbon is very close to 0 V (vs. Na/Na^+^), the simultaneous deposition of metallic Na is highly possible, resulting in hazardous accidents once the batteries are internally short‐circuited or exposed to air and water by damage. Based on this consideration, we choose Sb–SiC–C alloy composite as safer and higher‐capacity anodes to construct safer Na ion batteries.

In order to acquire the better electrochemical performance, the amount of FEC in the electrolyte was carefully optimized for Sb‐based anode materials (Figure S2, Supporting Information). The initial charge and discharge curves of Sb–SiC–C in TMP‐based electrolyte with 0%, 5 vol%, 10 vol%, and 15 vol% FEC are shown in Figure S2 (Supporting Information), exhibiting an initial coulombic efficiency of 22%, 60%, 68.3%, and 69.9%. Several reports showed that FEC additive causes a voltage shoulder around 0.7 V (vs Na/Na^+^) during the first discharge, corresponding to SEI formation at the anode surface.^64^, ^65^ In this work, we also observed this voltage shoulder in the discharge curve of the Sb–SiC–C anode in all FEC containing electrolyte. In contrast, the Sb–SiC–C anode in FEC free electrolyte displays a prolonged discharge plateau at 0.5 V with a low initial coulombic efficiency of 22%, due to a continuous decomposition of TMP. These results demonstrate that FEC can form compact SEI film, which effectively suppress the decomposition of TMP so as to greatly improve the coulombic efficiency of the Sb–SiC–C anode. Since the Sb–SiC–C anodes in TMP‐based electrolytes containing 10 vol% and 15 vol% FEC show similar high initial coulombic efficiency and electrochemical performance, the amount of FEC in electrolyte is optimized as 10 vol%.

**Figure 3:**
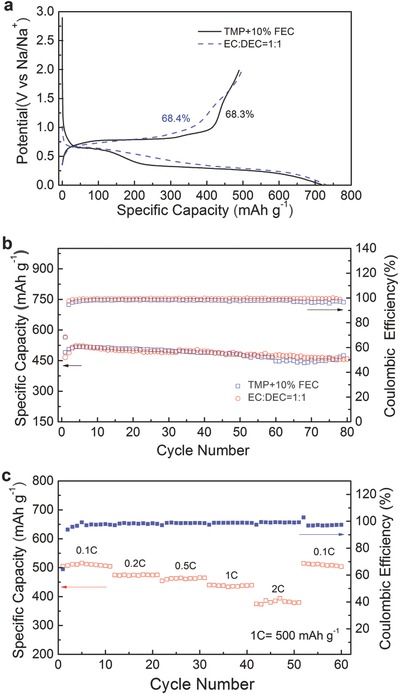
Electrochemical characterizations of Sb‐based anode: a) Initial charge/discharge curves. b) Cycling performance in 0.8 m NaPF_6_/TMP electrode with or without 10 vol% FEC and in carbonate electrolyte. c) Rate capability in the 0.8 m NaPF_6_/TMP + 10 vol% FEC electrolyte.

**Figure** **3**a compares the charge–discharge performances of the Sb–SiC–C anode in the nonflammable TMP electrolyte with 10 vol% FEC and in conventional carbonate electrolyte. The initial discharge and charge capacities of the Sb‐based anode in the TMP + 10 vol% FEC electrolyte is 717 and 489 mAh g^−1^ at a current density of 50 mA g^−1^, respectively, corresponding to an initial coulombic efficiency of 68.3%, which is almost equal to that (68.4%), observed in carbonate electrolyte (Figure 3a). The cycling performance of the Sb‐based anode in the TMP + 10 vol% FEC electrolyte is also shown in Figure 3b. It can be seen that the capacity retention of the Sb‐based anode is close to 94% over 80 cycles, similarly as stable as in the carbonate electrolyte. The coulombic efficiency of the Sb‐based anode increased rapidly from 68.4% at the first cycle to 99% in the a few cycles and then remained steadily at subsequent cycles, demonstrating an excellent reversibility of the Na‐storage reaction in the TMP‐based electrolyte. Besides, the Sb‐based anode can deliver a reversible capacity of 380 mAh g^−1^ at 2 C (1 C corresponds to a current density of 500 mA g^−1^), which is even higher than that of hard carbon at very low rate (270 mAh g^−1^),^28^, ^30^, ^31^ exhibiting a very high rate capability (Figure 3c). Therefore, the Sb‐based anode in the TMP + 10 vol% FEC electrolyte functions similar electrochemical performances as in conventional carbonate electrolytes, displaying excellent compatibility, which enable to be used as a safe anode in the nonflammable TMP‐based electrolyte.

### 2.3 **Electrochemical Performances of NaNi_0.35_Mn_0.35_Fe_0.3_O_2_ Cathodes in the TMP‐Based Electrolyte**

**Figure 4:**
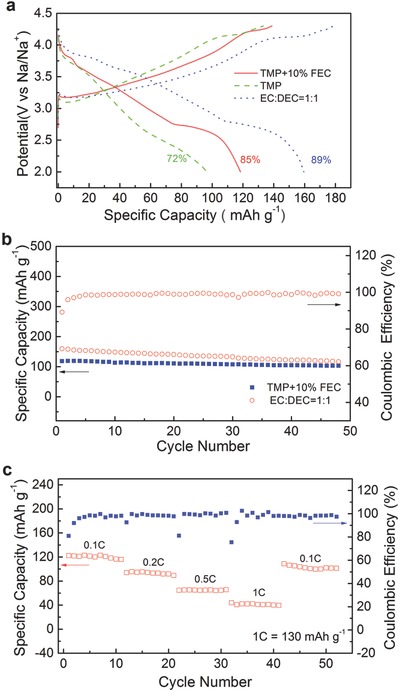
Electrochemical characterizations of NaNi_0.35_Mn_0.35_Fe_0.3_O_2_ cathode: a) Initial charge/discharge curves. b) Cycling performance in the 0.8 m NaPF_6_/TMP electrolyte with or without 10 vol% FEC electrolyte and in carbonate electrolyte. c) Rate capability in the 0.8 m NaPF_6_/TMP + 10 vol% FEC electrolyte.

**Figure** **4** compares the charge–discharge performance of NaNi_0.35_Mn_0.35_Fe_0.3_O_2_ cathode in different electrolytes. As displayed in Figure 4a, the NaNi_0.35_Mn_0.35_Fe_0.3_O_2_ cathode in the TMP + 10 vol% FEC electrolyte gives two pairs of charge/discharge plateaus at 4.1/3.8 V and 3.3/2.7 V, respectively, corresponding to the redox reaction of Ni and Fe ions, which are in good agreement with their CV features (Figure 2b). Although the charge–discharge curves of the NaNi_0.35_Mn_0.35_Fe_0.3_O_2_ electrode in the TMP + 10 vol% FEC electrolyte are similar to that observed in the carbonate electrolyte, the reversible capacity is much lower (120 than 160 mAh g^−1^) at a current density of 15 mA g^−1^. This could be possibly explained by that the low ionic conductivity and poor wettability of the TMP‐based electrolyte causes higher electrochemical polarization so as to lower capacity at voltage plateau of above 4.0 V (Figure 4a). However, the NaNi_0.35_Mn_0.35_Fe_0.3_O_2_ cathode in the TMP + 10 vol% FEC electrolyte exhibits comparable initial coulombic efficiency (85%) as that (89%) in the carbonate electrolyte, indicating that the decomposition of TMP + 10 vol% FEC at potential up to 4.3 V (vs Na/Na^+^) are insignificant. Whereas, the NaNi_0.35_Mn_0.35_Fe_0.3_O_2_ cathode in the TMP electrolyte without FEC shows lower reversible capacity (96 mAh g^−1^) and initial coulombic efficiency (72%), indicating FEC is also necessary for stable SEI film forming on cathode to alleviate the decomposition of TMP at high charge potential. The capacity retention of the NaNi_0.35_Mn_0.35_Fe_0.3_O_2_ cathode is close to 85% over 50 cycles at 0.1 C (1 C = 130 mA g^−1^) in the TMP + 10 vol% FEC electrolyte (Figure 4b), which is comparable to that (89%) in carbonate electrolyte. Figure 4c shows the rate cycling behavior of the NaNi_0.35_Mn_0.35_Fe_0.3_O_2_ cathode manipulated from 0.1 C to 1 C. The electrode delivers a reversible capacity of 120, 95, 65, and 40 mAh g^−1^ at the current rates of 0.1 C, 0.2 C, 0.5 C and 1 C (1 C = 130 mA g^−1^), respectively. Though the rate capability is lower than that in the carbonate electrolyte due to the low ionic conductivity of the TMP electrolyte, it is sufficient for normal energy storage applications. Certainly, additives with higher dipole moment and lower viscosity should be very helpful to enhance the ionic conductivity of the TMP‐based electrolyte and should be under investigation. Notably, the coulombic efficiency of the electrode rapidly increases from 85% at the first cycle to 95% at the third cycle and then maintains higher than 95% afterward, indicating that the electrolyte decomposition process only happens in the initial several cycles. Thus, the NaNi_0.35_Mn_0.35_Fe_0.3_O_2_ cathode also shows good electro­chemical compatibility with the TMP + 10 vol% FEC electrolyte, exhibiting sufficient reversible capacity, rate capability, and excellent cycling stability.

### 2.4 **Surface Chemical Characterization and Microscopy Analysis**

**Figure 5:**
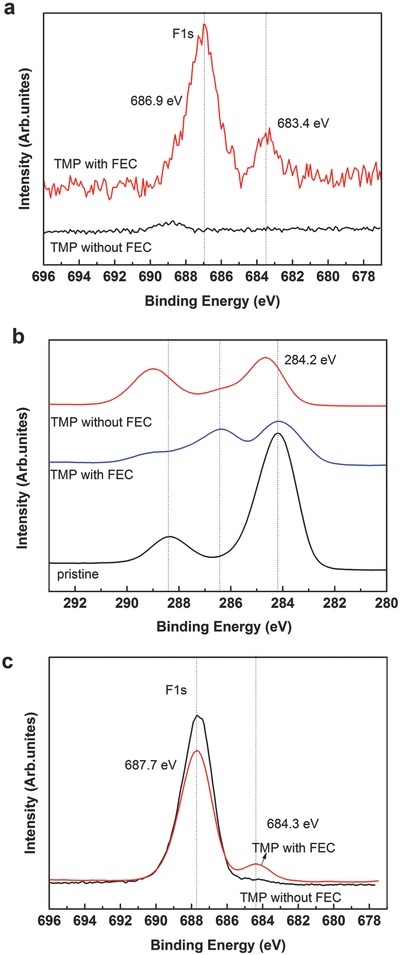
XPS results for elements a) F, b) C in the Sb–SiC–C electrodes after 10 cycles at 50 mA g^−1^ and c) F in the NaNi_0.35_Mn_0.35_Fe_0.3_O_2_ electrode after 10 cycles at 15 mA g^−1^ in TMP electrolytes with or without FEC.

In order to further understand the role of the FEC additive, the morphological changes and chemical compositions of the electrodes before and after 10 cycles were investigated (Figure S3, Supporting Information). As can be seen, the Sb‐based anode cycled in FEC‐free electrolyte for 10 cycles (Figure S3b, Supporting Information) present similar coarse surface as the fresh electrode (Figure S3a, Supporting Information), suggesting indiscernible SEI film is formed.^66^ In contrast, the surface of the Sb electrode cycled for 10 cycles in the FEC‐containing electrolyte is very fuzzy (Figure S3c, Supporting Information), suggesting the presence of SEI coating. The chemical compositions of the SEI films on the Sb anode and NaNi_0.35_Mn_0.35_Fe_0.3_O_2_ cathode are studied using X‐ray photoelectron spectroscopy (XPS) and shown in **Figure** **5**. In order to avoid the confusion of the resource of F signal, NaPF_6_ is replaced by NaClO_4_, which has no adverse effect on the electrochemical performance of the two materials. As can be seen in Figure 5a, the F1s spectra of the Sb anode cycled in the FEC‐free TMP electrolyte show no F signal at all, while the spectra in the FEC‐containing TMP electrolyte has a distinct peak at 686.8 eV and a small bulge at 683.4 eV, corresponding to some kinds of organic fluorides and NaF, respectively.^64^, ^66^, ^67^ Thus, the XPS spectra clearly demonstrate the SEI formation on the Sb surface by FEC decomposition. The C1s spectra of the Sb anodes are presented in Figure 5b. The cycled Sb anodes in TMP electrolytes with or without FEC show intense peaks around 284–286 and 288–290 eV, corresponding to the hydrocarbon, alkyl carbon, and alkoxy species.^64^, ^68^ Notably, the peaks in FEC‐containing electrolyte shift slightly to higher binding energy, probably induced by the presence of F‐containing alkyl carbon species due to the formation of SEI film by FEC decomposition on the surface of Sb anode.^64^ For the NaNi_0.35_Mn_0.35_Fe_0.3_O_2_ cathodes, both F1s spectra in electrolyte with or without FEC have a distinct peak at 687.7 eV (Figure 5c), corresponding to some organic fluorides from PDVF binder. It is noticed a new peak appears at 687.7 eV in FEC‐containing electrolyte, probably can be attributed to NaF.^67^ This phenomenon indicates that FEC decomposition also contributes to the SEI formation on the cathode. Thus, XPS results demonstrated that FEC in TMP electrolyte can form stable SEI layers on both NaNi_0.35_Mn_0.35_Fe_0.3_O_2_ cathode and Sb‐based anode, so as to effectively suppress the TMP decomposition, improve the initial efficiency and reversible capacity of the electrodes.

### 2.5 **Construction and Characterization of a Safer Na‐Ion Battery**

**Figure 6:**
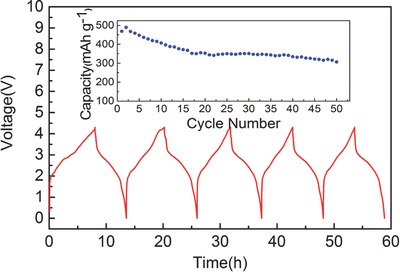
Charge/discharge curves and cycling performance of Sb/NaNi_0.35_Mn_0.35_Fe_0.3_O_2_ cells in the 0.8 m NaPF_6_/TMP + 10 vol% FEC electrolyte.

Based on the above results, we constructed a safer Na‐ion battery by coupling the Sb‐based anode and NaNi_0.35_Mn_0.35_Fe_0.3_O_2_ cathode, together with the nonflammable TMP + 10 vol% FEC electrolyte. The full cells were designed as an anode‐limited type (i.e., cathode capacity > anode capacity) to compensate the initial irreversible capacity of the Sb anode. **Figure** **6** shows the charge–discharge curves and cycling performance of the Sb/NaNi_0.35_Mn_0.35_Fe_0.3_O_2_ full cells in the TMP + 10 vol% FEC electrolyte at a constant current of 50 mA g^−1^. This full cell displays charge/discharge plateaus around 3.1 and 3.0 V, and initial reversible capacity of 489 mAh g^−1^ (based on the Sb anode), similar to the half cell (Figure 3a). The coulombic efficiency of the full cell increases from 58% at the first cycle to 98% after several cycles, suggesting that both Sb anode and NaNi_0.35_Mn_0.35_Fe_0.3_O_2_ cathode perform perfectly in the TMP + 10 vol% FEC electrolyte. The capacity of the full cell decreases slowly at the initial several cycles and then remains stable, which is different from stable cyclability observed from Sb and NaNi_0.35_Mn_0.35_Fe_0.3_O_2_ half batteries (Figure 3b and Figure 4b). This phenomenon is possibly resulted from an imperfect match of the coulombic efficiency between the anode and cathode during the initial charge–discharge cycles and needs to be further studied.

### 3 Conclusions

In summary, a safer sodium ion battery is constructed by using Sb‐based anode, NaNi_0.35_Mn_0.35_Fe_0.3_O_2_ cathode, and nonflammable TMP + 10 vol% FEC electrolyte. The TMP + 10 vol% FEC electrolyte totally cannot be ignited completely, exhibiting excellent thermal safety. The Sb‐based anode and NaNi_0.35_Mn_0.35_Fe_0.3_O_2_ cathode in the TMP + 10 vol% FEC electrolyte show high reversible capacity (490 and 120 mAh g^−1^ at first cycle) and capacity retention (94% and 86% after 50 cycles), suggesting excellent electrochemical compatibility with the TMP + 10 vol% FEC electrolyte. The good electrochemical performance mainly results from the stable SEI film formed by FEC decomposition on the anode and cathode, which alleviate effectively the unfavorable side reactions between the TMP and the electrode materials. As a result, the Sb/NaNi_0.35_Mn_0.35_Fe_0.3_O_2_ full cell demonstrates considerable charge–discharge capacity and cycling performance. Our study demonstrates for the first time that a safer sodium ion battery can be achieved using nonflammable organic phosphate electrolyte. We believe that through optimizing electrode materials, phosphate electrolyte, sodium salt, and film‐forming additive, nonflammable sodium‐ion batteries are promising to meet the high‐capacity and high‐safety requirements for energy storage applications.

## 4 Experimental Section

The TMP reagent used in this work was purchased commercially from Aladdin. Before preparing the electrolyte, TMP was first dehydrated with CaH_2_ under the atmosphere of inert gas for 12 h and then purified with a re‐distillation step under vacuum. A nonflammable electrolyte was obtained by mixing NaPF_6_ (Alfa Aesar), TMP and FEC to form 0.8 m NaPF_6_ in TMP with 10 vol% FEC. The fluoroethylene carbonate (FEC) was dehydrated with CaH_2_ under the atmosphere of inert gas for 12 h and then purified with a re‐distillation step under vacuum by oil pump.

Sb‐based anode material (Sb–SiC–C composite) was prepared by a high‐energy ball‐milling process.^63^ First, commercial Sb (99.0% purity, 74 μm, National Medicine Co., Ltd., Shanghai, China) and nano SiC (99.5% purity, 40–60 nm) was mixed in a 8:1 weight ratio and ball‐milled in a high‐energy mechanical mill (8000M mixer/mill, SPEX, USA) for 8 h. Then, the product was further ball milled with super P carbon (TIMCAL, Graphite & Carbon Inc.) in a planetary mill (QM‐1SP04, Nanjing, China) with the rotation speed of 200 rpm for 6 h. The weight ratio of super P to the total amount of Sb@SiC was designed as 1:9, so that the final composite composed of 80 wt% Sb, 10 wt% SiC, and 10 wt% super P. The weight ratio of milling balls to the powder materials in the planetary milling process was 20:1. All the milling processes were performed under argon atmosphere.

NaNi_0.35_Mn_0.35_Fe_0.3_O_2_ cathode material was prepared by a sol–gel method based on citric acid as described in previously report.^4^ A typical process is as following. The aqueous solution of stoichiometric sodium, iron, nickel, and manganese nitrates were added to critic acid solution^19^ and then stirred for 6 h at 70 °C into water bath. The resulting gel was dried at 120 °C for 24 h and precalcinated at 450 °C in air for 6 h to decompose the nitrate and eliminate the water. Finally, the powder precursor was ground and then calcined at 900 °C for 15 h in air atmosphere to obtain target materials.

The ionic conductivities of the selected electrolytes were measured on DDS‐307 (INESA Scientific Instrument Co., Ltd., Shanghai, China) at the temperature range of −20–40 °C. The morphological changes of the SiC–Sb–C electrode before and after cycling in FEC‐free and FEC‐containing electrolyte were characterized by scanning electron microscopy (FE‐SEM, ZEISS Merlin Compact VP, Germany). The composition on the surface of the electrode before and after cycling in FEC‐free and FEC‐containing electrolyte were characterized by spectrophotometer (XPS, Thermo Fisher ESCALAB 250Xi) with monochromatized Al Ka radiation (*hn* = 1486.6 eV).

The cyclic voltammetry (CV) were performed on CHI600C Electrochemical Analytical Instrument (Chenhua, Shanghai, China) in 0.8 m NaPF_6_ in TMP electrolyte with or without 10 vol% FEC by using microelectrode with a Pt disk of 0.5 mm diameter as working electrode, Na foil as both reference electrode and counter electrode.

The electrochemical performance measurement of the anode and cathode in the nonflammable electrolyte (0.8 m NaPF_6_ in TMP electrolyte with 10 vol% FEC) was carried out by using CR2016 coin cells. The Sb‐based anodes were prepared by mixing 80 wt% anode‐active materials (Sb–SiC–C), 10 wt% super P and 10 wt% poly(acrylic acid) (PAA) together and dissolving the electrode mixture into distilled water to form homogeneous slurry, then coating the electrode slurry on Cu foil. The positive electrodes consisted of 75 wt% NaNi_0.35_Mn_0.35_Fe_0.3_O_2_, 15 wt% acetylene black, and 15 wt% PVDF. Cell performance was achieved on a multi‐channel battery cycler (Land CT2001A, Wuhan, China) by assembling CR2016 coin cells of Na/Sb–SiC–C, Na/NaNi_0.35_Mn_0.35_Fe_0.3_O_2_ and Sb–SiC–C/NaNi_0.35_Mn_0.35_Fe_0.3_O_2_ in the glove box. These cells were cycled between a settled range of cell potential and current densities (Na/Sb–SiC–C, Na/NaNi_0.35_Mn_0.35_Fe_0.3_O_2_ and Sb–SiC–C/NaNi_0.35_Mn_0.35_Fe_0.3_O_2_). For comparison, a nonflammable electrolyte without FEC and conventional carbonate electro­lyte (0.8 m NaPF_6_ in EC‐DEC (1:1 vol)) were also used to measure the electrochemical performance of the materials.

## Supporting information

As a service to our authors and readers, this journal provides supporting information supplied by the authors. Such materials are peer reviewed and may be re‐organized for online delivery, but are not copy‐edited or typeset. Technical support issues arising from supporting information (other than missing files) should be addressed to the authors.

Supplementary
