# Supplementary material for: A Safer Sodium‐Ion Battery Based on Nonflammable Organic Phosphate Electrolyte
Source: Adv Sci (Weinh). 2016 Apr 23;3(9):1600066. doi: 10.1002/advs.201600066 (PMC5039966; doi:10.1002/advs.201600066)
Supplement: Supplementary file 1 — Supplementary [file ADVS-3-0m-s001.pdf]

Copyright WILEY-VCH Verlag GmbH & Co. KGaA, 69469 Weinheim, Germany, 2016.

ADVANCED SCIENCE
Open Access

Supporting Information

for *Adv. Sci.,* DOI: 10.1002/advs.201600066

A Safer Sodium-Ion Battery Based on Nonflammable Organic Phosphate Electrolyte

*Ziqi Zeng, Xiaoyu Jiang, Ran Li, Dingding Yuan, Xinping Ai, Hanxi Yang, and Yuliang Cao**

Supporting Information

A Safer Sodium-ion Battery based on Nonflammable Organic Phosphate Electrolyte

*Ziqi Zeng, Xiaoyu Jiang, Ran Li, Dingding Yuan, Xinping Ai, Hanxi Yang and Yuliang Cao**

Z. Q. Zeng, X. Y. Jiang, D. D. Yuan, Prof. X. P. Ai, Prof. H. X. Yang, Prof. Y. L. Cao
College of Chemistry and Molecular Sciences, Wuhan University, Wuhan 430072, China. E-mail: ylcao@whu.edu.cn
Dr. R. Li,
School of measurement and control technology and communication engineering, Harbin University of Science and Technology, Harbin 150080, China

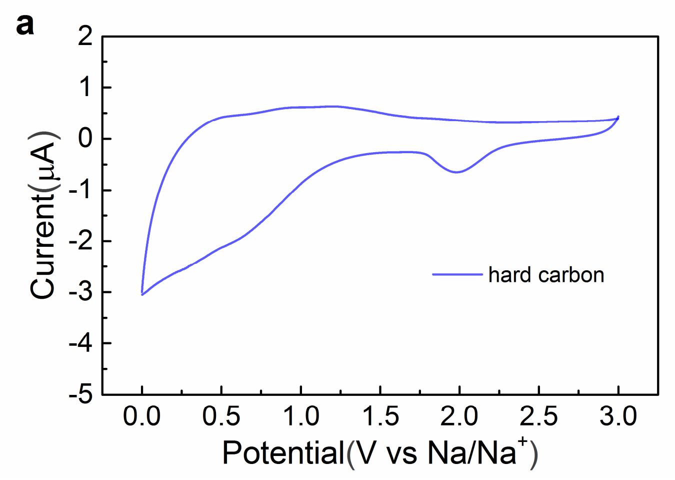

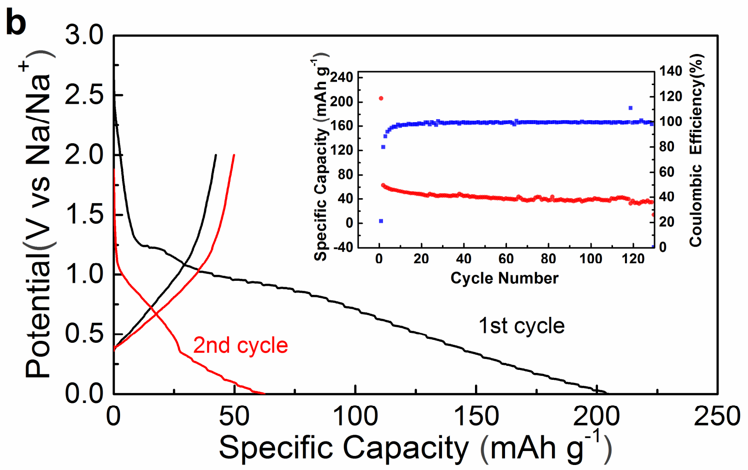

Figure S1 Electrochemical characterizations of hard carbon anode materials: (a) Cyclic voltammograms. (b) Charge/discharge curves in 0.8 M $NaPF_6$/TMP + 10 vol.% FEC. Inset: Cycling performance.

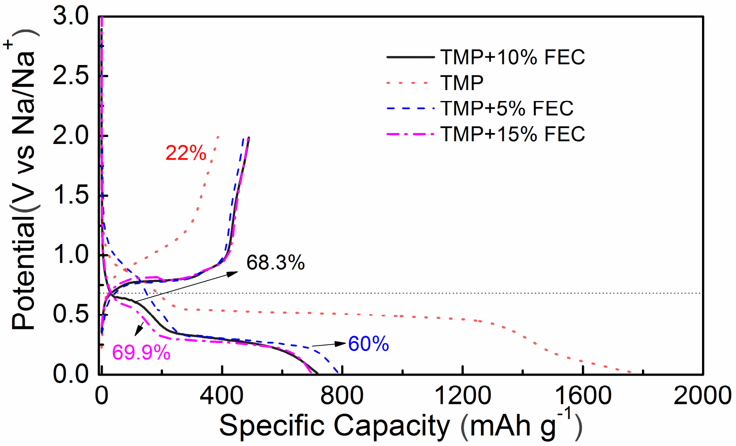

Figure S2 The initial charge/discharge curves of TMP electrolyte with 0%, 5 vol.%, 10 vol.% and 15 vol.% FEC at a current density of 50 mA g^{-1}.

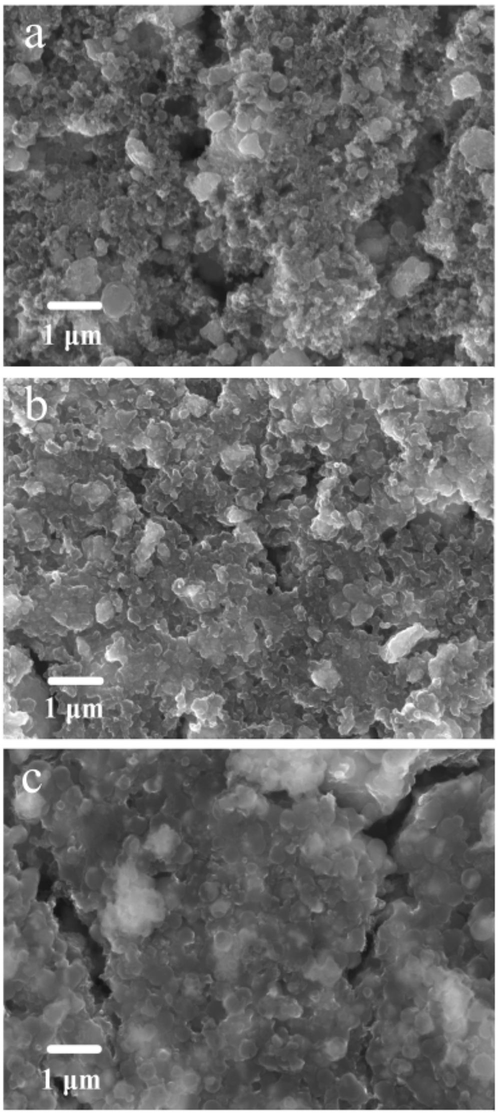

Figure S3 SEM images of the Sb-SiC-C electrode before (a) and after 10 cycles at 100 mA g^{-1} in FEC-free (b) and FEC-containing (c) $NaPF_6$/TMP electrolytes.
